# Recent Progress in Low-Power-Consumption Metal Oxide Semiconductor Gas Sensors

**DOI:** 10.3390/ma18214864

**Published:** 2025-10-24

**Authors:** Yu Zhang, Renbo Li, Ruqi Guo, Mingzhi Jiao, Gang Wang, Zhikai Zhao

**Affiliations:** 1 School of Safety Engineering, China University of Mining and Technology, Xuzhou 221116, China; zhangy74@cumt.edu.cn; 2 National & Local Joint Engineering Laboratory for Internet Application Technology in Mines, Xuzhou 221116, China; ts24060193p31@cumt.edu.cn (R.L.); ts24060009a31@cumt.edu.cn (R.G.); 3 School of Information and Control Engineering, China University of Mining and Technology, Xuzhou 221116, China

**Keywords:** MOS gas sensors, room temperature, MEMS, low power-consumption, methane, carbon monoxide, nitrogen dioxide, hydrogen

## Abstract

Metal oxide semiconductor (MOS) gas sensors offer several advantages, including low cost, high accuracy, and ease of miniaturization. Thus, they are excellent candidates for environmental monitoring and food spoilage detection applications, particularly in the safe Internet of Things field or for portable instruments. Typically, there are two general routes for realizing low-power-consumption MOS gas sensors: room-temperature MOS gas sensors or MEMS MOS gas sensors. The review focuses on the detection of four typical gases, namely methane, hydrogen, carbon monoxide, and nitrogen dioxide, systematically summarizing and analyzing the most recent results of low-power-consumption MOS gas sensors. The 2D materials, MOS composites, and 3D structured composites exhibit excellent room-temperature gas detection capabilities. The mechanism of the room-temperature gas sensors is also discussed in detail. Another route is MEMS MOS gas sensors. First, the progress of the micro-hotplate research is introduced. Then, several of the latest reported MEMS MOS gas sensors are shown and compared. The gas sensing mechanism of these MEMS MOS gas sensors is also given. The paper will provide a valuable guide for researchers in the MOS gas sensor field, particularly for those working towards low-power-consumption MOS gas sensors.

## 1. Introduction

As the economy grows, more hazardous gases can be found in the outdoors or in factories. Methane can be found in coal mines or the kitchen. It can give rise to an explosion or severe combustion in the event of a leak. Furthermore, methane contributes much more to the greenhouse effect than carbon dioxide. Hydrogen is a promising fuel considering its high efficiency and cleanliness. However, it has a relatively wide explosion concentration range (from 4% to 75.6%). Additionally, nitrogen dioxide and carbon monoxide are two highly toxic gases that can cause severe harm to humans when humans are exposed to high concentrations for a short period. Thus, accurate monitoring of the above four gases is necessary to ensure city safety. Several gas sensor technologies, including optical, electrochemical, metal oxide semiconductor resistive, and catalytic combustion gas sensors, have been utilized for gas safety monitoring. Metal oxide semiconductor (MOS) resistive gas sensors offer advantages such as low cost, ease of miniaturization, high accuracy, and a low detection limit, making them widely applicable in safety monitoring [1]. However, MOS gas sensors generally consume high power due to heater integration, casting a challenge for safe Internet of Things applications or portable devices. Low-power-consumption MOS gas sensors are gaining increasing attention, particularly in light of the power constraints in many applications. Core technologies for low-power-consumption gas sensors can be categorized into two main types: room-temperature solutions and MEMS-based designs [2,3,4]. Both approaches achieve energy efficiency through material innovation and structural-material optimization, respectively, accelerating the development of low-power-consumption gas sensors. The rest of the paper is organized in the following way: First, the progress of room-temperature gas sensors is introduced, with focus on the materials innovation and working mechanism discussion; Secondly, the recent development of MEMS gas sensors is described, with emphasis on micro-hotplate design and sensing materials; Thirdly, the challenge and prospects of low-power-consumption MOS gas sensors are discussed. Ultimately, the conclusion is presented regarding the current state of the art of the two solutions for low-power-consumption MOS gas sensors.

## 2. Room-Temperature Gas Sensors

Traditional metal oxide gas sensors require operation above room temperature, with heaters being the primary source of power consumption. These devices also pose safety risks due to the detection of flammable and explosive gases, as well as issues such as time-consuming preheating and significant material heat loss. In contrast, room-temperature gas sensors operate without heating, offering advantages such as no preheating required, low power consumption, minimal material heat loss, a simple structure, and reduced high-temperature hazards. These outstanding benefits have attracted considerable attention from researchers, who have conducted extensive studies on room-temperature gas sensors, with a particular focus on detectors for methane (CH_4_), hydrogen (H_2_), carbon monoxide (CO), and nitrogen dioxide (NO_2_). NO_2_ is a typical oxidative gas, while the other three gases are reductive. The four gases are chosen due to their wide existence in environmental and industrial scenarios.

### 2.1. Room-Temperature CO Sensors

**Figure 1:**
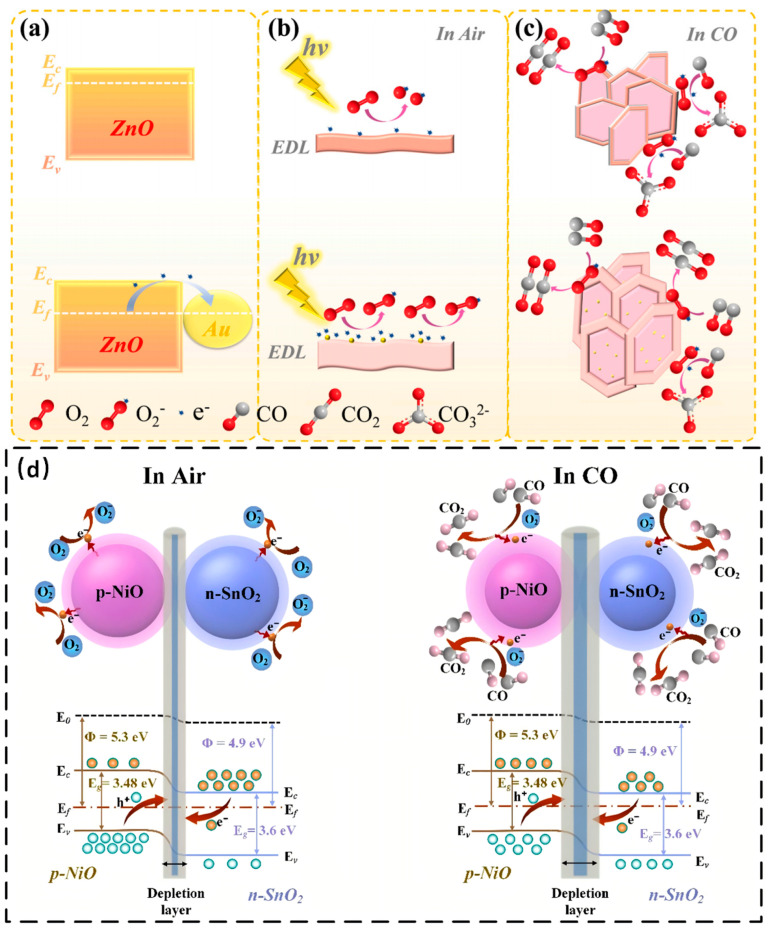
(**a**) The energy band structure of ZnO and Au-ZnO [8]; (**b**,**c**) The mechanism of Au nanoparticles on Au/ZnO and the reaction of CO on the surface of samples [8]; (**d**) diagram of CO-sensing mechanism from p-NiO/n-SnO_2_ composites [10].

Carbon monoxide is a rather lethal gas, causing severe death in a few minutes when the concentration is higher than 1200 ppm. A review by He et al. has summarized and compared many relevant works before 2024 [5]. Here, we will introduce some excellent work on room-temperature CO sensors published after 2024. Noble metal decoration can decrease the working temperature of metal oxide semiconductors. For example, Pd-CuO-SnSe_2_ can respond to 200 ppm CO in 13 s [6]. It is reported that 1 wt% Pt-SnO_2_ nanoceramics can react with 400 ppm CO at room temperature, yielding a response value of 2427 [7]. Wu et al. reported that 0.1Au-ZnO can respond to 100 ppm CO at room temperature in 61 s with a value of 139.75 [8]. The sensing mechanism of Au-ZnO can be explained in two aspects: the Schottky barrier and the catalytic function of Au. Since the work function of Au is lower than that of the conduction band of ZnO, electrons at the conduction band of ZnO will flow into the surface of Au and form Schottky barriers, enriching the number of electrons in Au and enlarging the surface depletion layer of ZnO. Schottky barriers will amplify resistance variations and inhibit electron-hole recombination, as shown in Figure 1a. Au with a higher electron density can enhance oxygen adsorption and dissociation, as shown in Figure 1b,c. Au will facilitate oxygen adsorption when the Au-ZnO is in the air. In CO, Au from the composite enhances the adsorption and dissociation of CO, thereby increasing the CO surface reaction and sensing. CuO-SnO_2_ nanotubes were synthesized by assembly on the carbon nanotube templates, followed by subsequent calcination [9]. The optimal sensor can respond to 300 ppm in 56 s. The response value can be as high as 1.34, with an ultra-low detection limit of 159 ppb. Xie et al. successfully synthesized SnO_2_-NiO through the MOF template [10]. The sensor exhibits a response value of 5.48 toward 100 ppm CO in 56 s, with a detection limit as low as one ppm CO. The enhanced sensing performance of SnO_2_-NiO can be attributed to the p-n heterojunction effect, as seen in Figure 1d. When the material is exposed to air, oxygen will trap electrons from the conduction band, increasing the number of hole carriers and forming a hole stacking layer, which will reduce the resistance of the sensor. When the sensor is exposed to CO, the trapped electrons recombine with the holes, causing the hole stacking layer to become thinner and the depletion layer to become thicker, which in turn increases the sensor resistance, as shown in Figure 1d.

Furthermore, NiO-Ti_3_C_2_T_x_ (MXene) composite was reported to respond to 400 ppm CO in only 8 s, with a response value of 1.3 [11]. The excellent sensing properties of NiO-Ti_3_C_2_T_x_ are due to the superior characteristics of Ti_3_C_2_T_x_. The outer surface of Ti_3_C_2_T_x_ can be functionalized by different groups such as -F, -OH, or =O [12,13]. These groups can serve as attachment sites for surface-functional molecules, thereby enhancing the selectivity and sensitivity of the gas sensors. Ti_3_C_2_T_x_ with -F and -OH groups have significantly different band gaps as well (0.72 eV for -F and 1.07 eV for -OH). In addition, Ti_3_C_2_T_x_ has a room temperature conductivity of up to 10,000 S cm^−1^ [14,15].

As shown in Table 1, carbon-based composites exhibit good sensitivity and response speed to CO. For example, MWCNT/SnO_2_ composites can react to 300 ppm CO in 5 s with a response value of 1.80 [16]. MWCNT and SnO_2_ will form a p-n junction when the two come in contact, since MWCNT is a p-type material and SnO_2_ is a typical n-type semiconductor. Furthermore, rGO-wrapped SnS_2_ nanospheres can respond to 10 ppm CO in 11 s as well, with a response value as high as 10 [17]. On the one hand, a heterojunction between n-type SnS_2_ and p-type rGO will enhance the CO response; on the other hand, the presence of numerous defect sites on the rGO surface will further increase the CO response.

### 2.2. Room-Temperature NO_2_ Sensor

**Figure 2:**
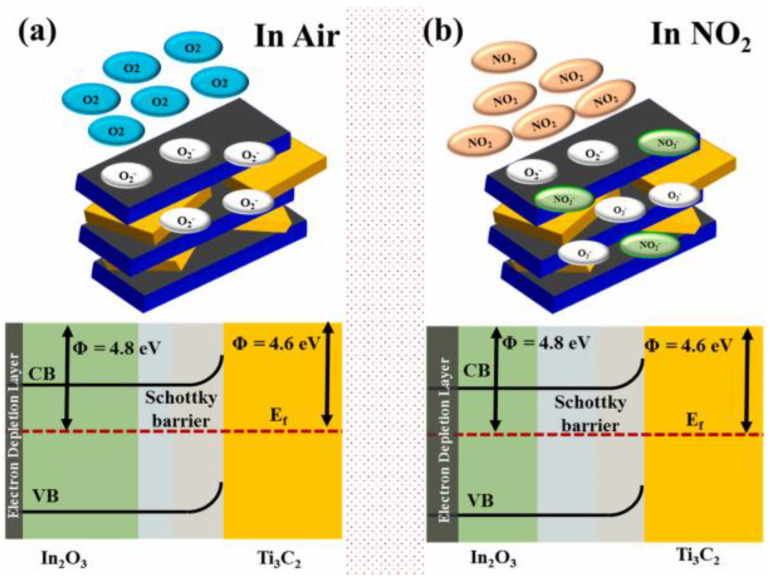
Diagram of gas-sensing mechanism and band structure of the In_2_O_3_/Ti_3_C_2_: (**a**) The surface nad band structure of In_2_O_3_/Ti_3_C_2_ in air [22]; (**b**) The surface nad band structure of In_2_O_3_/Ti_3_C_2_ in NO_2_ [22].

Recently, 2D materials such as reduced graphene oxide composites, Mxene, and transition metal dichalcogenides have been reported to detect NO_2_ with excellent performance, even at room temperature. For example, rGO/CuO nanoflakes can respond to 5 ppm NO_2_ at room temperature in only 6.8 s, with a response value of 1.26 [20]. rGO/In_2_O_3_ was found to have an ultrahigh response value of 1177 toward one ppm NO_2_ [21]. In_2_O_3_/Ti_3_C_2_ nanosheets can respond to 100 ppm NO_2_ in 18 s, with a response value of 371.19 [22]. The mechanism for room-temperature NO_2_ sensing from In_2_O_3_/Ti_3_C_2_ is attributed to the Schottky barrier, as shown in Figure 2.

A detailed comparison of the room-temperature NO_2_ sensing performance of 2D materials, as recently reported, is presented in Table 2. Even though In_2_O_3_/Ti_3_C_2_ nanosheets can respond to 100 ppm NO_2_ in 18 s, the recovery time is as long as 95 s. The prolonged recovery time of the materials is attributed to intense interactions between NO_2_ and the In_2_O_3_/Ti_3_C_2_ nanosheets, which pose challenges for the desorption of NO_2_ gas.

Except for 2D materials, traditional metal oxides and sulfides are being explored for NO_2_ sensing, offering high and fast responses. Notably, MoO_3_@CoMoO_4_@CoMoS_3.13_ can respond to 40 ppm NO_2_ in only 1.1 s, with a response value as high as 35.42 [35]. A possible NO_2_ sensing mechanism was proposed as follows: (1) NO_2_ is first absorbed onto the surface layer (CoMoO_4_@CoMoS_3.13_) of the nanocomposite containing S-modified vacancies to contact with Co sites. (2) The electron is then sucked by the NO_2_ molecule and reacts with the chemisorbed oxygen (2NO_2_ (gas) + O_2_^−^ (ads) + 2e^−^ →2NO_3_^−^ (ads)). (3) A full recovery of the resistance is accompanied with the removal of NO_2_ from the sensing system, realizing the return of the electron to material. Recently, Zhang et al. found that ZnInS_4_ can respond to 10 ppm NO_2_ in only 2 s, with a response value of 3.28 [36]. The excellent room-temperature NO_2_ sensing properties of ZnInS_4_ may result from the presence of rich sulfur vacancies. The breakneck response/recovery speed is due to the open structure of ZnIn_2_S_4_/MIL-68(In), which promotes the adsorption and diffusion of NO_2_ molecules.

**Figure 3:**
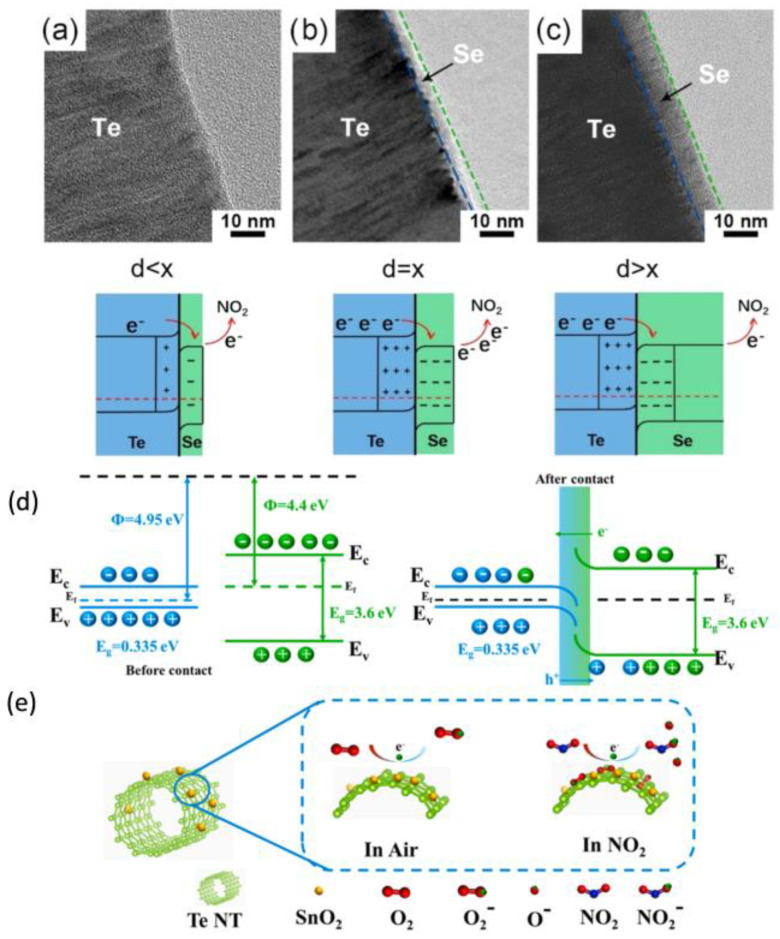
TEM images (top) and the corresponding heterojunction band structure (bottom) of Te@Se with different thicknesses of Se [37]: (**a**) Te@Se-1, (**b**) Te@Se-2, and (**c**) Te@Se-3; Schematic diagrams illustrating the gas-sensing mechanism of Te NT/SnO_2_ [38]: (**d**) band structures of Te and SnO_2_, (**e**) sensing process of the TeNT/SnO_2_-based sensor.

Tellurium (Te), a 2D elemental material, is promising for NO_2_ detection due to its suitable band structure for gas adsorption and charge mobility. However, Pure Te materials have poor stability due to their high activity, which limits their application in gas sensor fields. Core−shell 2D Te@Se heterostructures are prepared using a solvothermal method [37]. The Te@Se heterostructures with a thickness of 4–6 nm of Se demonstrate an exceptionally high response of 7.22 to 1 ppm of NO_2_ at room temperature, with ultrafast response/recovery times of 10 s and 30 s, respectively. The room-temperature NO_2_ sensing performance of Te@Se depends strongly on the thickness of the Se shell, as seen in Figure 3a–c. Three cases may happen: (1) When the Se shell is too thin, the electron accumulation layer in Se is smaller than the theoretical thickness, leading to a minimal electron migration effect and resistance modulation ability (as shown in Figure 3a); (2) When the thickness of the Se shell exceeds that of the electron accumulation layer, the electrons in the accumulation layer must pass through the Se shell to bind with NO_2_ (Figure 3c), resulting in lower electron mobility in Se. Consequently, the efficient transfer of accumulated electrons to NO_2_ is hindered. (3) The resistance modulation effect from electron migration is maximized only when the thickness of the Se shell matches that of the electron accumulation layer, as seen in Figure 3b. As a result, the highest NO_2_ response can be achieved in this case. Furthermore, SnO_2_-decorated Te nanotubes are found to respond to 600 ppb NO_2_ in 32 s, with a response value of 1.33 [38]. The mechanism for enhanced sensor performance can be attributed to Schottky barriers, as shown in Figure 3d. When the material is exposed to NO_2_, the adsorbed NO_2_ can form adsorbed NO_2_^−^ through either capturing electrons from SnO_2_ decorated Te or reacting with surface O_2_^−^, as illustrated in Figure 3e. A detailed comparison of related materials can be found in Table 3.

### 2.3. Room-Temperature H_2_ Sensor

**Figure 4:**
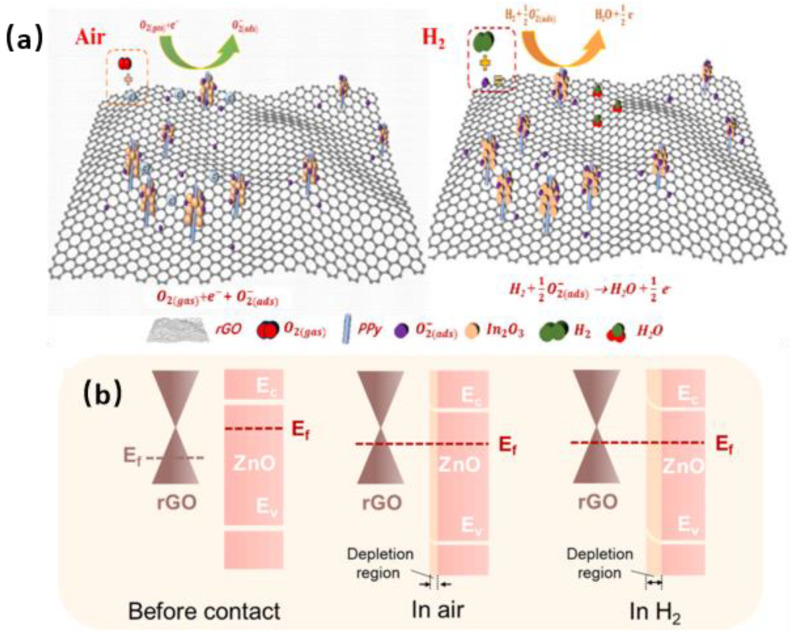
Gas-sensitive response mechanism of different 3D materials: (**a**) In_2_O_3_-rGO-Ppy [45]; (**b**) Cactus-like ZnO@3D rGA [46].

H_2_ is attracting increasing attention due to its excellent properties, including high efficiency, cleanliness, and renewability. However, it has a relatively wide explosion limit (4~75%), causing a significant threat to human and city safety. Room-temperature H_2_ sensing is particularly meaningful for avoiding the heating risk associated with the sensor itself [42,43,44]. Three-dimensional (3D) structure nanomaterial composites have been investigated for rapid room-temperature H_2_ sensing. For example, Chen et al. successfully synthesized a 3D In_2_O_3_-rGO-PPy composite aerogel using a hydrothermal method [45]. It can respond to 1000 ppm H_2_ in 13 s with a response value of 11.6. The mechanism is as follows: The oxygen gas will form chemisorbed oxygen ions (O_2_^−^) when oxygen molecules are adsorbed on the surface of the 3D In_2_O_3_-rGO-Ppy at room temperature. After H_2_ exposure, the oxygen ions on the surface react with H_2_ to form H_2_O, as shown in Figure 4a.

Furthermore, Cactus-like ZnO@three-dimensional reduced graphene oxide aerogels (Cactus-like ZnO@3D rGA) respond to 10,000 ppm H_2_ in 15 s, achieving a response value of 81.07 [46]. The enhanced sensing performance is attributed to the p-n heterojunction between ZnO (n-type with a work function of 4.25 eV) and rGO (p-type with a work function of 4.86 eV), as shown in Figure 4b. When ZnO is in contact with rGO, electrons from ZnO will be transferred to rGO. In contrast, the holes in rGO will be transferred to ZnO in the opposite direction. The superior room temperature sensing behavior of ZnO-rGO toward H_2_ can be attributed to two reasons: on the one hand, rGO can increase the conductivity of the composites and form a heterojunction with ZnO; on the other hand, 3D rGO possesses a pore structure, which facilitates the adsorption and desorption of gases. Additionally, the porous structure can increase the number of active sites for surface gas reactions. A summary of the performance of related materials is shown in Table 4.

### 2.4. Room-Temperature CH_4_ Sensor

**Figure 5:**
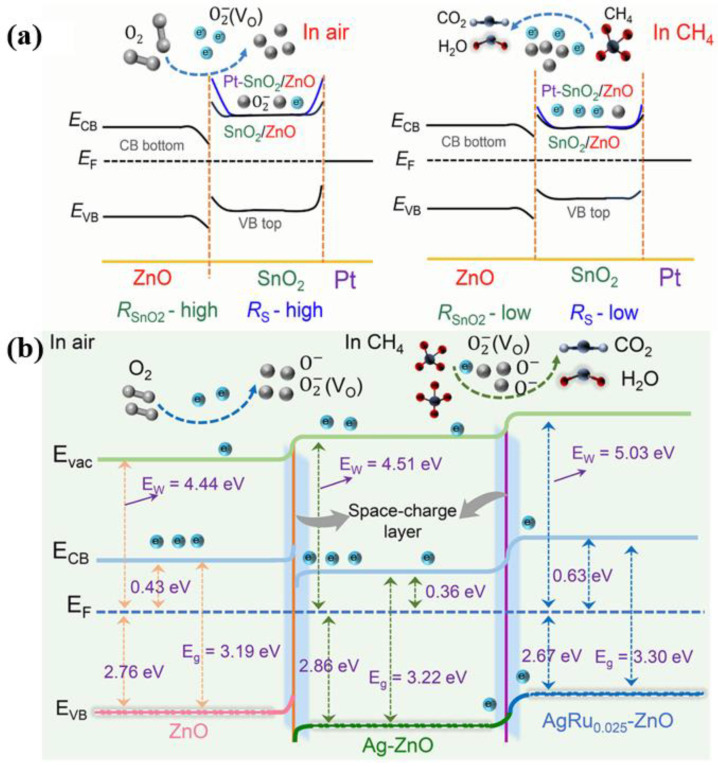
(**a**) Band structure and methane sensing mechanism of: (**a**) Pt-SnO_2_/ZnO [52]; (**b**) AgRu/ZnO [54].

Li et al. created Pt-doped SnO_2_/ZnO bilayer structures showing CH_4_ response at room temperature [52], attributed to the combined effects of n-n heterojunction and Schottky barrier, as depicted in Figure 5a. Optimal sensitive materials: 1.0Pt-SnO_2_/ZnO exhibits prolonged response and recovery times exceeding 100 s for a 2000 ppm CH_4_ concentration. Yang et al. designed In_2_O_3_-ZnO/laser-induced graphene composites for room-temperature CH_4_ sensors, achieving a 27.48% response at 500 ppm CH_4_ with response/recovery times of 48/169 s, respectively, and a detection limit as low as 3 ppm [53]. Li et al. prepared Ag-Ru co-doped ZnO nanorod arrays, enabling CH_4_ detection at room temperature [54]. As seen in Figure 5b, the work function of ZnO is 4.44 eV, while that of Ag/ZnO and AuRu_0.025_/ZnO is 4.51 and 5.03 eV, respectively. The Fermi level in Ag-ZnO contributes to the enhanced oxygen vacancy density, and that in AuRu_0.025_-ZnO proves the role of p-type lattice defects. The localized contact between Ag-ZnO/AuRu_0.025_-ZnO supports the experimental result that the electrons are transferred from the Ag−Ru co-doping site to the nearby Ag-doping site, reducing Ag^+^ to Ag^0^ in AgRu_0.025_-ZnO. The localized contact between ZnO/Ag-ZnO reveals that more electrons accumulate in the Ag-doping sites, which act as activity sites for O_2_ chemical adsorption.

Detailed sensing information of related materials is presented in Table 5. To be noteworthy, UV light activation can be an effective route to enhance the methane sensing performance at room temperature [55,56]. For example, AuAg/ZnO can respond to 5000 ppm CH_4_ in 5 s, with a response of 62.61 [56]. The conductivity of ZnO will increase under UV illumination since the electrons of ZnO will be excited from the valence band to the conduction band. Oxygen molecules will trap photogenerated electrons adsorbed on the ZnO surface, forming reactive oxygen species and creating an electron-depletion layer. Upon CH_4_ exposure, CH_4_ molecules react with oxygen ions, causing electrons to return to ZnO and reducing resistance.

To summarize, despite significant advancements in related research, several practical application shortcomings persist for this type of sensor, including susceptibility to humidity, prolonged response times, reduced sensitivity to low gas concentrations, and poor selectivity for target gases. It is not competitive enough in scenarios with high requirements on stability and sensitivity.

## 3. MEMS Gas Sensor

**Figure 6:**
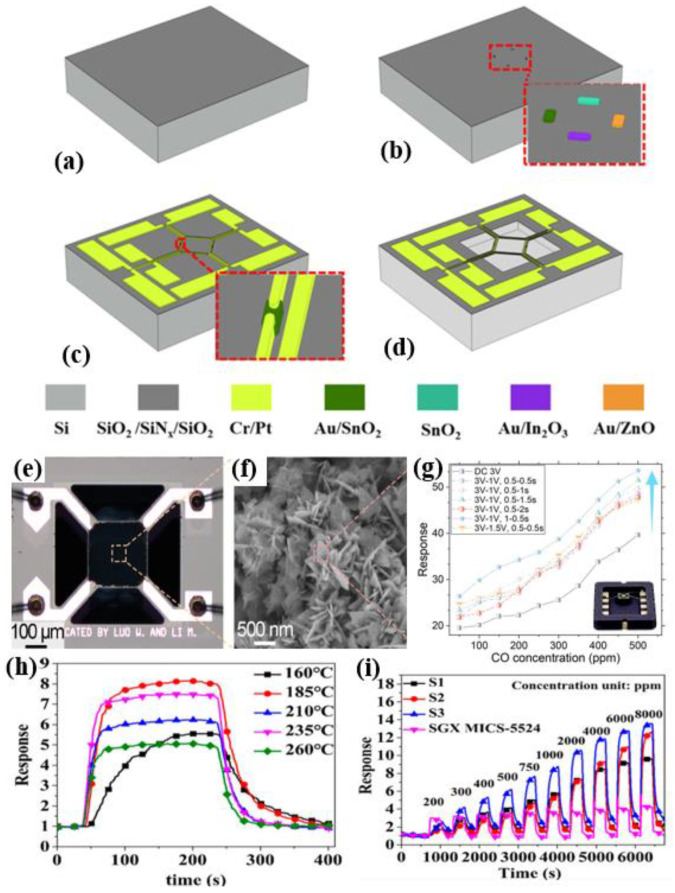
(**a**–**d**) Fabrication steps of the proposed gas sensor cell [65]: (**a**) Supporting layer by LPCVD; (**b**) Four sensing material deposition and patterning; (**c**) Heater and detecting electrodes patterning; (**d**) Dry-etching processes for releasing suspended structures. (**e**) Optical image of the SnO_2_ nanosheet on the MEMS hotplate [66]; (**f**) SEM of the SnO_2_ nanosheet on the MEMS hotplate [66]; (**g**) Sensor response to CO under different modes (constant temperature vs. pulse heating [66]). (**h**) Response of Pd-loaded SnO_2_ nanofiber sensors at 2000 ppm H_2_ exposure under varying temperatures [68]; (**i**) Dynamic response curves of three S1, S2, and S3 labeled SnO_2_ nanofiber MHP gas sensors to H_2_ [68].

MEMS sensors, based on microelectromechanical systems (MEMS) technology, are typically compact (1 μm to 1 mm in size) and feature unique manufacturing processes that enable the easy construction of gas sensing arrays or grids. These sensors offer several significant advantages, including high sensitivity, rapid response times, low power consumption, and easy integration. By developing and refining sensitive materials with varying characteristics, they can effectively detect different gases. Hadano et al. developed a MEMS catalytic combustion CH_4_ gas sensor featuring platinum-loaded catalysts as reference elements to enhance methane selectivity, while improving CH_4_ sensitivity through structural modifications, such as enhancements to the micro-thermal plate design [62]. Yang et al. fabricated a quadri-lateral MEMS MOS gas sensor cell with four different sensitive materials based on a complete semiconductor process, as seen in Figure 6a–d. Jang et al. designed a MEMS catalytic combustion micro-gas sensor for leak detection [63]. Measuring just 3 mm × 3 mm, this sensor achieves an output voltage of 0.29 mV at 1158 ppm CH_4_ and 104 mW of input power, demonstrating both low power consumption and excellent selectivity.

MEMS catalytic combustion sensors exhibit relatively high power consumption and are susceptible to poisoning effects. In contrast, MEMS metal oxide sensors offer several advantages, including lower-power consumption, higher sensitivity, lower detection limits, greater material modification potential, and improved stability. Yan et al. designed an intelligent MEMS sensor based on FeCoNi oxidation of an entropy alloy, which reduces power consumption via a pulse-heating mode [64]. At 150 °C, it shows excellent selectivity for H_2_ and CO with detection limits of 0.3 ppm and 0.29 ppm, respectively. Yang et al. made a quadrilateral MEMS metal oxide gas sensor cell with four different sensitive materials based on a complete semiconductor process, as shown in Figure 6a–d [65]. Li et al. proposed a MEMS CO sensor based on SnO_2_ nanosheets, as shown in Figure 6e,f [66]. Figure 6g compares the sensor’s response to CO under constant-temperature and pulse-heating modes, revealing a response of 34.3 at 200 ppm CO in pulse mode—1.56 times that in the constant-temperature mode—with approximately 19.8 mW of power consumption. Luo et al. developed a single MEMS sensor based on SnO_2_ nanosheets [67]. At 370 °C, this sensor achieves a response of 297.7 ppm to H_2_, with power consumption as low as 26.6 mW in pulse-heating mode. Wu et al. proposed a H_2_ sensor based on Pd-loaded SnO_2_ nanofibers, demonstrating an excellent response to 200 ppm H_2_ at 185 °C (Figure 6h), with a power consumption of only 9.1 mW [68]. Figure 6i compares the response values of self-made S1, S2, and S3 sensors with those of the commercial MICS-5524 sensor at 185 °C for H_2_ concentrations ranging from 200 to 8000 ppm, showing that the self-made sensors outperform the commercial sensor at high concentrations. Li et al. proposed a MEMS differential thermoelectric stack hydrogen sensor with response and recovery times of 14 s and 10 s, respectively, at 400 ppm H_2_, and a power consumption of only 61 mW [69].

In summary, MEMS metal oxide gas sensors achieve rapid response to target gases with low power consumption in gas detection by integrating micro-structured designs with suitable sensitive materials. Current research on low-power-consumption MEMS metal oxide gas sensors primarily focuses on two aspects: First, optimizing the sensor’s micro-hotplate structure—high thermal conductivity silicon-based microstructures reduce heat loss and lower power consumption while maintaining appropriate temperatures. Second, improving sensitive materials through nanostructuring to enhance specific surface area for gas adsorption efficiency, or constructing composite metal oxide systems to optimize selectivity and stability while reducing baseline drift during prolonged operation. These synergistic advancements collectively reinforce the core advantages of MEMS sensors—“low power consumption, high responsiveness, and miniaturization”—forming an essential foundation for functional and performance breakthroughs.

### 3.1. Research Progress of MEMS Micro Hotplate

A micro hotplate (MHP) is a crucial component in MEMS gas sensors. It is based on silicon microfabrication technology, and its basic structure includes a silicon substrate, a suspended distributed support film, and a heater electrode. The power consumption of MHP is mainly affected by its materials and structure.

**Figure 7:**
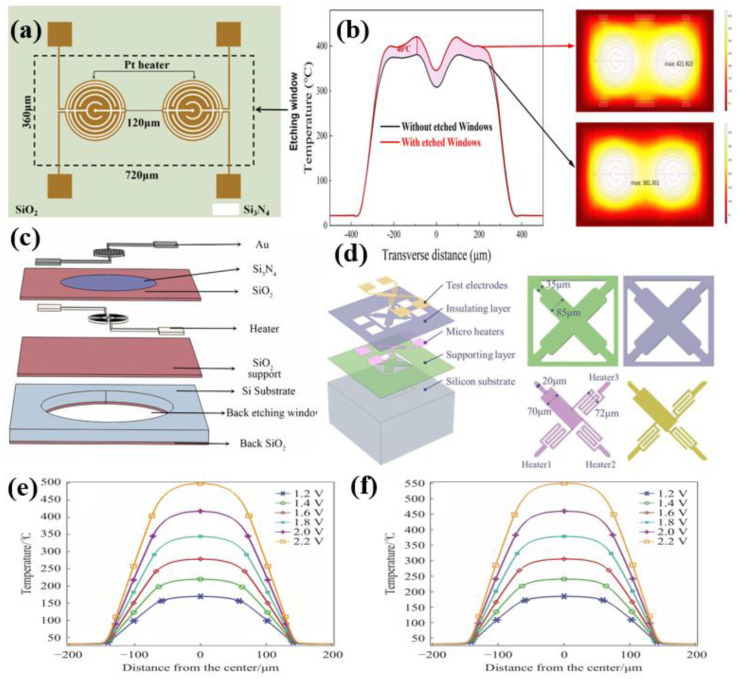
(**a**) Schematic diagram of the planar configuration of the thermal quenching heater MHP [70]; (**b**) Temperature distribution of MHP with and without etched windows [70]; (**c**) Structural schematic of Si_3_N_4_-SiO_2_ composite dielectric layer MHP [72]; (**d**) Three-micro-thermal-plate integrated array structure MHP [73]; The temperature distributions under different heating voltages for structures 1 and 2 designed by our research group are shown as follows: (**e**) Structure 1 [74]; (**f**) Structure 2 [74].

Yang et al. proposed a novel multi-heater MHP design based on thermal coupling [70]. Figure 7a shows the planar schematic of a dual-heater MHP, where heaters with a 100 μm radius achieve excellent temperature uniformity at 300 °C. The thermal coupling effect significantly reduces MHP power consumption, saving approximately 4.68 mW compared to single-heater configurations. Figure 7b illustrates the temperature distribution in MHPs with and without etched windows. The etched window design leverages air’s superior thermal insulation, increasing peak temperatures by about 40 °C while effectively reducing heat loss. Zhang et al. developed an MHP for wafer-level packaging of MEMS gas sensors using an ONO composite layer as support [71]. By analyzing different heater electrode shapes and cantilever beam materials, they found that serpentine electrodes produced the most uniform temperature distribution. The optimized MHP exhibits lower power consumption and enables the high-density integration of multiple independent, temperature-controlled MHPs.

Wei et al. introduced a planar MHP design combining Si_3_N_4_-SiO_2_ lateral composite dielectric layers with annular heaters (Figure 7c) [72]. The composite dielectric layer effectively reduces lateral heat conduction losses and mitigates mechanical deformation in planar microheat plates. Zhang et al. created a novel MHP array structure integrating three MHPs per suspended region (Figure 7d) [73]. This configuration enhances integration density and achieves independent temperature control. Using COMSOL simulations, they optimized the platinum heater thickness and adjusted the cantilever and electrode widths, effectively reducing crosstalk interference. We conducted finite-element analysis of two serpentine MHP platinum electrode configurations. Structure 1 features identical heating electrode width and spacing, while Structure 2 maintains uniform spacing but exhibits a gradually increasing micro-heater width from both sides toward the center. Figure 7e,f display temperature variations under different heating voltages for structures 1 and 2. Through COMSOL simulation analysis, it was found that under equivalent power consumption, Structure 2 demonstrates slightly higher maximum central temperature than Structure 1, while achieving lower power consumption at the same temperature levels [74].

Multiple researchers have optimized MHP structures by integrating heaters, selecting appropriate support layers and electrode configurations, introducing composite dielectric layers, and designing arrays. These optimizations have demonstrated significant improvements in power efficiency, stability, selectivity, and integration density, providing diverse approaches for related optimization and applications. However, limitations remain: precise control of multi-heater thermal crosstalk may compromise detection accuracy; etching windows and composite dielectric layers could reduce structural strength, increasing fracture risk; cross-interference in arrays remains challenging to eliminate; and some designs involve complex processes that increase mass-production costs. These issues highlight the need to strike a better balance between structural stability, thermal interference control, and process simplification.

### 3.2. MEMS MOS Sensors Based on Various Sensitive Materials

In view of the problems encountered by MEMS gas sensors in practical applications, such as low response values and poor selectivity, this paper will summarize and analyze a series of MEMS gas sensors with excellent performance by further optimizing the sensitive materials for CO, NO_2_, CH_4_, and H_2_.

#### 3.2.1. MEMS MOS CO Sensors

Carbon monoxide (CO), a primary hazardous gas in coal mines, originates from the spontaneous combustion of coal in mined-out areas and the incomplete burning of coal dust during mine gas explosions. This highly toxic, combustible substance can rapidly cause fatal consequences. Low-power-consumption CO sensors serve as critical safeguards for safe coal mining operations and form the essential foundation for distributed wireless sensing technologies. Comparison of the sensing performance of various MEMS MOS CO sensors can be found in Table 6.

**Figure 8:**
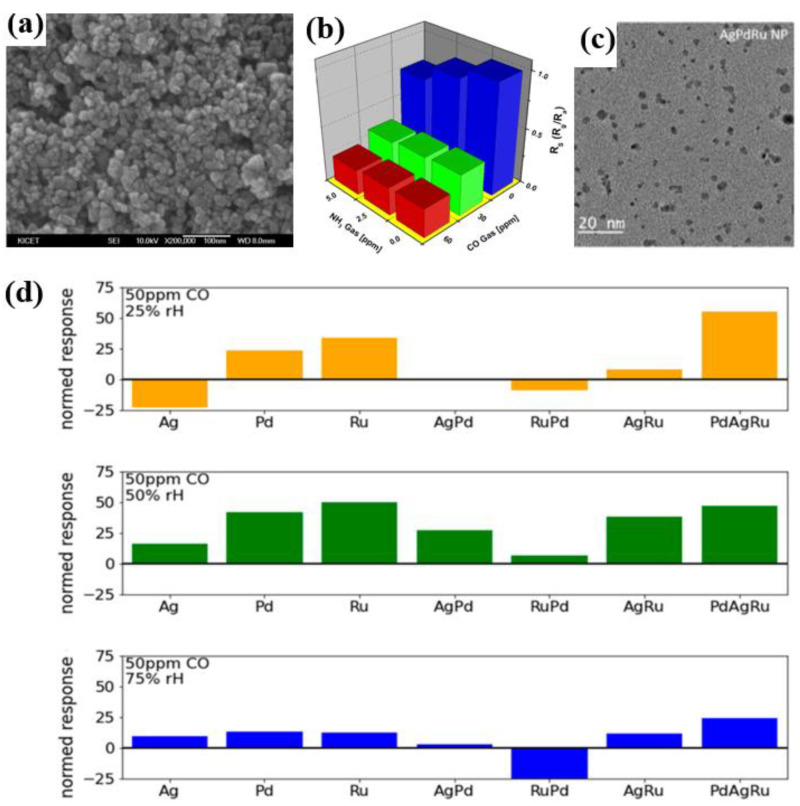
(**a**) SEM image of Pd-SnO_2_ nanomaterials [75]; (**b**) 3D bar chart of gas sensitivity characteristics of Pd-SnO_2_ sensors in CO-NH_3_ gas system [75]; (**c**) TEM image of the three-metal PdAgRu structures [77]; (**d**) Normalized response of different SnO_2_ composites toward 50 ppm CO at various humidity levels [77].

In recent years, MEMS-based CO sensors have garnered significant attention in the field. Kim et al. synthesized Pd-SnO_2_ nanoscale powders as CO gas sensors using the sol–gel method, as shown in Figure 8a [75]. In CO-NH_3_ mixed gases, CO exhibits significantly higher sensitivity than NH_3_, with Pd catalyzing CO adsorption and reactions that dominate the changes in sensitivity. The CO response is highly humidity-dependent, as illustrated in Figure 8b. Moon et al. developed a MEMS CO gas sensor by co-precipitating 10 wt% WO_3_ and 0.5 wt% Pt-doped SnO_2_ nanosheets [76]. This sensor consumes approximately 15 mW of power and detects CO at concentrations as low as 2 ppm. It shows a response value of about 1.31 at 20 ppm CO, with response and recovery times of 91 s and 134 s, respectively. Egger et al. developed a MEMS gas sensor based on SnO2 films, utilizing Ag, Pd, Ru, and their combinations as catalysts to enhance CO sensitivity while mitigating humidity-induced cross-sensitivity [77]. The sensor operates effectively at 200 °C and responds to CO and hydrocarbon mixtures ranging from 5 to 50 ppm. As shown in Figure 8c, the AgRu- and AgPd-functionalized sensors exhibit high CO sensitivity with low humidity cross-sensitivity. In contrast, the tri-metal PdAgRu-functionalized sensor exhibits the strongest CO response (Figure 8d). Vanmathi et al. optimized the fabrication parameters for Al-doped TiO_2_ films via radio-frequency magnetron sputtering [78]. The increased surface area provides more active sites for gas absorption, significantly altering the material’s resistivity. The sensor has a detection limit of 55% at a CO concentration of 200 ppm.

**Table 6:** Comparison of the sensing performance of various MEMS MOS CO sensors.

Sensing Material	Working Temperature (°C)	Conc. (ppm)	Response Value (R_g_/R_a_ or R_a_/R_g_)	Response/ Recovery Time (s)	Reference
10 wt%WO_3_/0.5 wt%Pt/SnO_2_ NS	400	20	1.31	91/134	[76]
Ag/Pd/Ru/SnO_2_	200	50	1.67	-/-	[77]
Al doped TiO_2_	400	200	2.22	-/-	[78]
Pd/SnO_2_ nanopowder	300	60	0.23	-/-	[75]

#### 3.2.2. MEMS MOS NO_2_ Sensors

Recently, MEMS MOS sensors have been explored for NO_2_ sensing, due to their high response, fast detection speed, and ease of integration. For example, Hsueh first synthesized Co_3_O_4_ nanoparticles (NPs) through ultrasonic wave grinding and then decorated the Co_3_O_4_ with Au NPs [79]. The Au/Co_3_O_4_-NPs MEMS gas sensor exhibits higher sensitivity to NO_2_ than to other gases (SO_2_, NH_3_, CO) at an optimal operating temperature of 136 °C. Comparison of the sensing performance of various MEMS MOS NO_2_ sensors can be found in Table 7.

**Figure 9:**
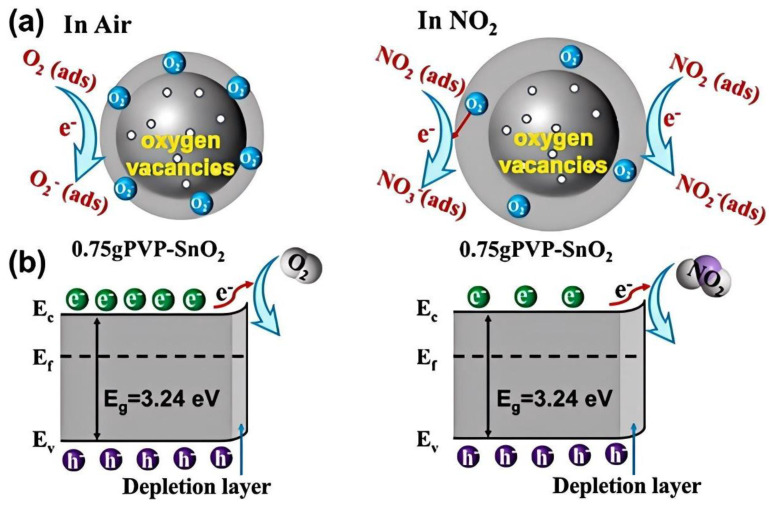
Gas sensing mechanism of oxygen-enriched SnO_2_ NPs before and after exposure to NO_2_ [83]: (**a**) Diagram of the gas-sensing mechanism; (**b**) Energy band structure and electron transfer.

CuO nanowires (NWs) can be produced through RF sputtering and integrated with a MEMS structure to form a MEMS sensor [80]. The sensor can detect 500 ppb NO_2_, with a response value of 1.63 at 131 °C. ZnO nanomaterial was deposited on a ceramic MHP by a microextruder [81]. The MEMS sensor has a response value of 41.6 toward 10 ppm NO_2_ at 200 °C. Meanwhile, Xu et al. obtained hierarchically porous ZnO by annealing Zeolitic Imidazolate Frameworks (ZIF-90) at 450 °C [82]. The optimal MEMS sensor can detect 10 ppm NO_2_ in 9 s with a high response. In addition, tiny SnO_2_ NPs with enriched oxygen vacancies are synthesized through the hydrothermal method assisted by PVP. The obtained SnO_2_ NPs are employed in MEMS gas sensors, which can have a response value of 14.5 toward 500 ppb NO_2_ at 102 °C in 45.9 s [83]. The superior gas-sensing performance of the SnO_2_ NO_2_ sensor can be attributed to the effect of oxygen vacancies, which will introduce multiple midgap states or donor energy levels between the valence and conduction bands of the sensing material, accelerating the excitation of hot electrons into the conduction band. This process facilitates the adsorption of more NO_2_ molecules onto the surface, where they rapidly capture electrons and subsequently participate in redox reactions, ultimately enhancing the gas-sensing performance, as shown in Figure 9a,b. Furthermore, doping with an appropriate amount of N not only increases the number of O_2_^−^ species, providing additional active sites for NO_2_ adsorption, but also enhances the electron-transfer rate, thereby improving carrier mobility in tin dioxide.

#### 3.2.3. MEMS MOS H_2_ Sensors

H_2_ is a highly flammable gas with an extensive explosive limit (4–75% in air). A leak mixed with air can easily ignite upon exposure to open flames or static electricity, potentially causing explosions or fires. H_2_ sensors enable real-time concentration monitoring and trigger alarms when concentrations approach hazardous thresholds, preventing safety incidents. In recent years, MEMS hydrogen sensors have achieved remarkable advancements in research.

Comparison of the sensing performance of various MEMS MOS H_2_ sensors can be found in Table 8.

**Figure 10:**
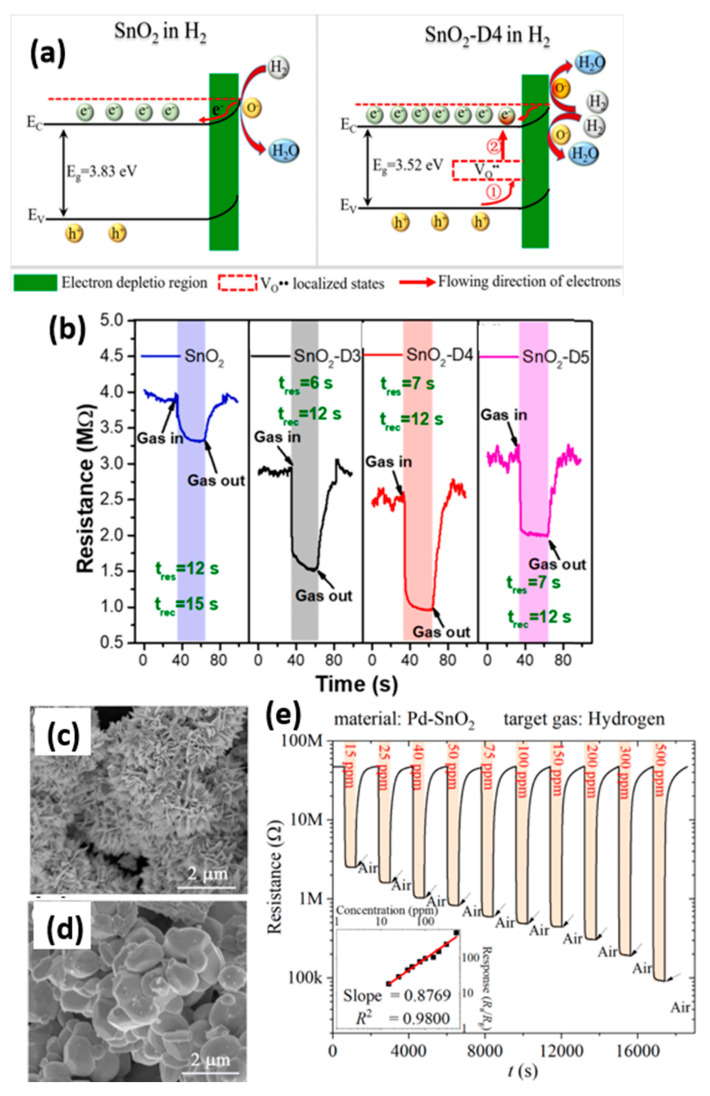
(**a**) Schematic diagrams of electronic structures and gas-sensitive response mechanisms for SnO_2_ and SnO_2_-D4 under H_2_ environment [86]; (**b**) The dynamic responses of different SnO_2_ samples, SnO_2_-D3/SnO_2_-D4/SnO_2_-D5 refers to SnO_2_ calcinate at 300/400/500 °C in H_2_ atmosphere [86]; (**c**) SEM image of Pd-SnO_2_ [87]; (**d**) SEM image of Pd-WO_3_ [87]; (**e**) The dynamic responses of Pd-SnO_2_ [87].

Zhang et al. developed a Pt-modified 0.4% Nb-doped TiO_2_-nanoplate MEMS hydrogen sensor that achieves 12.3 Hz response at room temperature for 1000 ppm H_2_, with response and recovery times of 31 s and 270 s, respectively, demonstrating excellent selectivity, stability, and repeatability [84]. He et al. created a reduced graphene oxide (rGO)-modified Nb-doped TiO_2_-nanoplate MEMS H_2_ sensor, where 10% rGO and 0.8% Nb-doped TiO_2_-nanoplates showed a 2.5 Hz response at 100 °C for 1000 ppm H_2_ with response/recovery times of 32.5/58 s [85]. Luo et al. [86] introduced an oxygen vacancy-enhanced SnO_2_ (SnO_2_-D) MEMS H_2_ sensor, where D4 modification altered the SnO_2_ microstructure while regulating electronic states and gas adsorption reactions, optimizing target gas adsorption, dissociation, and electron transfer to enhance gas-sensitive response performance, as shown in Figure 10a. At the optimal operating temperature of 250 °C, the SnO_2_-D4 sensor demonstrates a response value of 2.3 to 6 ppm H_2_, surpassing the original SnO_2_ (1.13). The response time and recovery time for six ppm H_2_ are approximately 7 s and 12 s, respectively, as shown in Figure 10b. Li et al. proposed a gas sensor array by integrating Pd-SnO_2_ nanoflower clusters with F-WO_3_ microparticles on low-power-consumption microthermal plates, as illustrated in Figure 10c,d [87]. Operating at 300 °C (17 mW power consumption), it exhibits high sensitivity and low cross-sensitivity to eight gases, including H_2_ and ammonia, as well as their mixtures. The Pd-SnO_2_ nanoflower cluster exhibits an excellent linear response across H_2_ concentrations ranging from 15 to 500 ppm, as shown in Figure 10e.

#### 3.2.4. MEMS MOS CH_4_ Sensors

**Figure 11:**
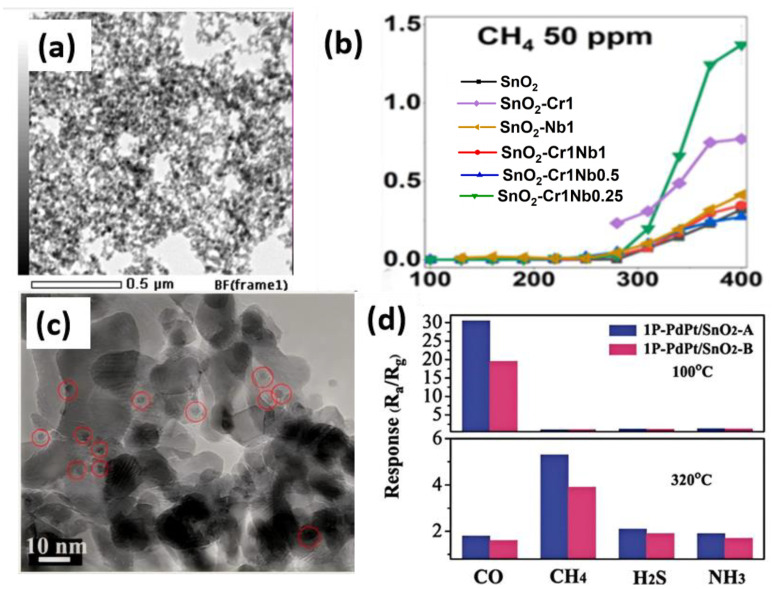
(**a**) TEM image of SnO_2_-Cr1Nb1 material [88]; (**b**) Time-dependent response of gas sensors [88]; (**c**) TEM image of 1P-PdPt/SnO_2_-A, red dots refer to PtPt bimetal [90]; (**d**) Response spectra of 1P-PdPt/SnO_2_-A to different gases at 100/320 °C conditions [90].

CH_4_, being a flammable and explosive gas, can easily cause fires or explosions, making real-time concentration monitoring crucial. The research on MEMS CH_4_ sensors has attracted increasing attention. Sagitova et al. synthesized Nb and Cr-doped SnO_2_ gas sensing materials via flame spray pyrolysis [88]. Figure 11a shows the TEM image of SnO_2_-Cr1Nb1 material, where hetero-doping reduced the response attenuation by half compared to pure SnO_2_, with a response range of 100–400 °C for CH_4_ pairs, as seen in Figure 11b. Niu et al. [89] fabricated MOS gas sensors based on Si/SiO_2_-doped flexible fiber substrates, achieving CH_4_ detection through the in situ synthesis of Co-doped ZnO nanorods on the fiber surfaces. The sensor exhibited a maximum response of 16% at 1000 ppm CH_4_ under an optimal operating temperature of 50 °C, with a power consumption of 3.2 mW/mm^2^ and response/recovery times of approximately 350 s/106 s, respectively. Li et al. modified SnO_2_ nanosheets with PdPt bimetallic nanoparticles enriched in Pd shells and Pt cores to construct MOS gas sensors [90]. Figure 11c displays the TEM image of the 1P-PdPt/SnO_2_-A material, which exhibits temperature-dependent dual selectivity, responding at 320 °C, with a peak response value of 5.2 for 1000 ppm CH_4_. Figure 11d shows the gas response of 1P-PdPt/SnO_2_ at 100/320 °C, demonstrating high selectivity for CH_4_. Murata et al. developed MEMS gas sensors containing 7.5 at% Pt/SnO_2_ thin-film catalysts [91]. The dispersed Pt atoms in this sensor maintain a stable SnO_2_ lattice configuration. Under reducing gas and 703 K reaction conditions, minimal oxygen loss from the surrounding lattice occurs without Pt aggregation. The oxygen deficit is readily restored by air oxidation, ensuring high methane sensitivity and a long service life, with detection capability up to 12,500 ppm CH_4_. A detailed comparison of different MEMS CH_4_ sensors is presented in Table 9.

## 4. Challenges and Prospects

Room-temperature gas sensors have attracted increasing attention in recent years, particularly due to their safety requirements and low power consumption. However, humidity and response speed are the primary concerns. Humidity effect can be alleviated through several routes: (1) surface decoration through functional materials, such as a catalytic filter or graphite [92,93]; (2) temperature modulation method [94]; (3) algorithm correction [95]. Surface decoration can provide reasonable protection against water vapor, though it may affect response speed if the coating is too thick. It will incur additional costs due to the addition of a functional substance. Algorithm correction has now become a hot topic in the humidity suppression area [96]. It has been shown in the previous section that fast gas response dynamics can be achieved by carefully tuning the material component and structure, as shown in [41,45].

The selectivity problem appears to be a universal issue for all MOS sensors, whether they operate at room or elevated temperatures, regardless of whether they use MEMS technology. It is due to the sensor’s surface reaction-based principle. Advanced algorithms, combined with sensor array design, can be a suitable solution for this issue [97]. There are two general routes for data processing algorithms: machine learning and deep learning. Machine learning algorithms have better Interpretability than deep learning ones, but they require manual feature extraction and are easily influenced by environmental noise and fluctuations. Deep learning algorithms have high tolerance to environmental fluctuations and high recognition accuracy. It can automatically extract deep features from the data, despite typically having more complex network structures and poor interpretability [98]. Recently, Convolutional neural networks, ResNet, LSTM, and Transformer algorithms have been applied to gas recognition, achieving ideal results [99,100,101]. Furthermore, multi-task learning frameworks have been investigated for the dual tasks of gas classification and concentration regression [102,103,104,105]. Temperature modulation is another way to address the problem of selectivity [106,107]. Recently, our group also investigated the temperature modulation method and combined it with an FPGA to achieve the classification of seven different types of gases [108].

The stability of MOS gas sensors is very critical for their practical application. A recent review provides several routes for improving the stability of MOS gas sensors [109]. Firstly, p-type material may be superior in humidity resistance [110]. It is due to the surface metal cations’ effect, i.e., facilitating strong oxygen adsorption and redox cycles, thereby inhibiting interference from water molecules. Secondly, hydrophobic modification with PDMS or rare-earth oxides can improve the interaction of water molecules [111,112].

## 5. Conclusions

This paper focuses on low-power-consumption metal oxide gas sensors for secure IoT applications, presenting research advancements in both room-temperature and MEMS gas sensors. Room-temperature gas sensors that operate without heating feature low power consumption and simple structures; however, they suffer from humidity sensitivity, slow response times, limited sensitivity to low-concentration gases, and limited selectivity. MEMS gas sensors excel in high sensitivity, rapid response, low power consumption, and ease of integration. Their low-power-consumption operation is achieved through optimized microthermal plate structures and improved sensitive materials, demonstrating outstanding performance in detecting CH_4_, H_2_, NO_2_, and CO. However, MEMS sensors face several challenges, including difficulties controlling thermal crosstalk, risks to structural integrity, and high production costs due to the complexity of their manufacturing processes. Future efforts should focus on enhancing room-temperature sensor performance (especially in selectivity and speed), promoting collaborative innovation in MEMS sensor structures and materials, and advancing intelligent, integrated solutions tailored for practical applications. A selectivity problem exists in both types of MOS gas sensors due to their working principles. Recent progress in advanced algorithms can provide an ideal solution for the selectivity problem.

## Figures and Tables

**Table 1:** Comparison of the performance of various room-temperature CO-sensing materials.

Sensing Material	Conc. (ppm)	Response Value (R_g_/R_a_ or R_a_/R_g_)	Response/Recovery Time (s)	Reference
Pd/CuO Nanorods/SnSe_2_ Nanoflower	200	1.53	13/58	[6]
1 wt% Pt/SnO_2_ nanoceramics	400	2427	-/-	[7]
0.10Au-decorated ZnO nanosheets	100	139.75	61/61	[8]
Au-Loaded WS_2_/SnO_2_	1	12.6	-/-	[18]
MOF-derived SnO_2_/NiO	50	5.48	56/4	[9]
CuO/SnO_2_ Hollow-Sphere	300	1.40	-/-	[5]
NiO/Ti_3_C_2_T_x_	400	1.43	8/16	[11]
PANI/Ti_3_AlC_2_/CeO_2_	500	1.17	347/-	[19]
MWCNTs/SnO_2_	300	1.80	5/7	[16]
rGO wrapped SnS_2_ nanosphere	10	10	11/10	[17]

**Table 2:** Comparison of the room-temperature NO_2_ sensing performance of various 2D materials.

Sensing Material	Conc. (ppm)	Response Value (R_g_/R_a_ or R_a_/R_g_)	Response/Recovery Time (s)	Reference
rGO/Bi_2_S_3_	1	9.8	22/106	[23]
rGO/CuO	5	11.04	10/110	[24]
rGO/CuO nanoflakes	5	1.26	6.8/55.1	[20]
vacancies-rich SnO_2_-RGO	1	5.8	95/385	[25]
rGO/In_2_O_3_	1	1177	675/559	[21]
8 wt% GO-mediated In_2_O_3_	1	1021	413/182	[26]
In_2_O_3_/Ti_3_C_2_ nanosheets	100	371.19	18/95	[22]
Ti_3_C_2_T_x_	10	1.14	-/-	[27]
Ti_3_C_2_T_x_-sphere-like CuO	50	1.57	13.5/20.9	[28]
Ti_3_C_2_-I	120	1.15	90/105	[29]
Ti_3_C_2_T_x_/WS_2_	1	1.15	-/-	[30]
V_2_CT_x_/SnS_2_	5	2.49	4.8/4.7	[31]
MoSe_2_-WS_2_	0.05	1.60	68.9/65.7	[32]
GaSe_0.58_O_0.42_	6	7.75	48/378	[33]
MoSe_2_/0.5–Co_3_O_4_	0.05	1.26	139/20	[34]

**Table 3:** Comparison of the room-temperature NO_2_ sensing performance of various non-2D materials.

Sensing Material	Conc. (ppm)	Response Value (R_g_/R_a_ or R_a_/R_g_)	Response/ Recovery Time (s)	Reference
MoO_3_@CoMoO_4_@CoMoS_3.13_	50	35.42	1.1/-	[35]
Te@Se Core−Shell	1	7.22	10/30	[37]
ZnInS_4_	10	3.28	2/3.7	[36]
Hollow Co_3_O_4_ nanocages in NiO cilia	100	47.4	1.3/9.6	[39]
UV-activated p-type CuCrO_2_	250	3.60	107/300	[40]
SnO_2_ decorated Te nanotubes	0.6	1.33	32/109	[38]
Au@In_2_S_3_/In_2_O_3_	100	20.7	12/27	[41]

**Table 4:** Comparison of the room-temperature H_2-_sensing performance of various materials.

Sensing Material	Conc. (ppm)	Response Value (R_g_/R_a_ or R_a_/R_g_)	Response/Recovery Time (s)	Reference
flower-like In_2_O_3_/SnS_2_	1000	3.43	-/-	[47]
Mace-like In_2_O_3_@ZnO microtubules	10,000	6.67	178/338	[48]
5.0Pd@ZnO	200	1.53	-/-	[49]
Pd-Mg alloy thin films	500	1.13	85/360	[50]
3D In_2_O_3_@rGO@PPy aerogel	1000	11.6	13/29	[45]
Cactus-like ZnO@3D rGA	10,000	81.07	15/27	[46]
MoSe_2_ -WSe_2_	25	1.60	16/30	[51]

**Table 5:** Comparison of the room-temperature CH_4_ sensing performance of various materials.

Sensing Material	Conc. (ppm)	Response Value (R_g_/R_a_ or R_a_/R_g_)	Response/Recovery Time (s)	Reference
Pt-SnO_2_/ZnO	2000	3.73	150/147	[52]
In_2_O_3_−ZnO/Laser-Induced Graphene	500	1.38	48/169	[53]
Ag−Ru Co-doped ZnO Nanorods	800	1.8	-/-	[54]
Mulberry-Like ZnO/SnO_2_ Hierarchical Structure	100	1.27	163/89	[57]
Photo-activated Au-modified ZnO microsphere	5000	4.65	-/-	[58]
UV-activated ZnO spheres	1000	10.18	6/134	[55]
UV-activated AuAg/ZnO	5000	62.61	5/-	[56]
NiO/ZnO	5000	8.61	32/182	[59]
MWCNT with MOF (PCN-14)	50,000	-	120/-	[60]
MWCNT/Pd	100	1.36	20/25	[61]

**Table 7:** Comparison of the sensing performance of various MEMS MOS NO_2_ sensors.

Sensing Material	Temperature (°C)	Conc. (ppm)	Response Value (R_g_/R_a_ or R_a_/R_g_)	Response/Recovery Time (s)	Reference
Au-Co_3_O_4_ NPs	136	0.1	1.34	84/68	[79]
CuO NWs	110	0.5	2	-/-	[80]
ZnO nanopowder	200	10	41.6	41/-	[81]
Hierarchically porous ZnO	190	10	3.42	9/26	[82]
Oxygen-enriched SnO_2_ NPs	102	0.5	14.7	45.9/178.2	[83]

**Table 8:** Comparison of the sensing performance of various MEMS MOS H_2_ sensors.

Sensing Material	Working Temperature (°C)	Conc. (ppm)	Response Value (R_g_/R_a_ or R_a_/R_g_)	Response/Recovery Time (s)	Reference
Pt/Nb-doped TiO_2_ nanoplate	40	1000	12.3	30/270	[84]
rGO/Nb-doped TiO_2_-nanoplate	100	1000	2.5	32.5/58	[85]
PdNPs@Al_2_O_3_	200	400	-	14/10	[69]
oxygen vacancy-enhanced SnO_2_	250	6	2.3	7/12	[86]
Pd-SnO_2_ nanoflower clusters with F-WO_3_ microparticles	300	15	18.7	10/22.5	[87]

**Table 9:** Comparison of the sensing performance of various MEMS MOS CH_4_ sensors.

Sensing Material	Working Temperature (°C)	Conc. (ppm)	Response Value (R_g_/R_a_ or R_a_/R_g_)	Response/Recovery Time (s)	Reference
Nb and Cr-doped SnO_2_	400	50	3.35	-/-	[88]
Co-doped ZnO nanorods	50	1000	1.19	350/106	[89]
PdPt/SnO_2_	320	1000	5.2	-/-	[90]
7.5 at% Pt-SnO_2_ thin-film	430	12,500	-	-/-	[91]

## Data Availability

No new data were created or analyzed in this study. Data sharing is not applicable to this article.
